# An unusual case of subacute combined degeneration due to nitrous oxide abuse, which relapsed after bariatric surgery: A case report

**DOI:** 10.1097/MD.0000000000030442

**Published:** 2022-09-02

**Authors:** Wei Chen, Zhihua Si, Yanping Bi, Bing Yang

**Affiliations:** a Department of Gastroenterology, Shuguang Hospital, Affiliated to Shanghai University of Traditional Chinese Medicine, Shanghai, China; b Department of Neurology, the First Affiliated Hospital of Shandong First Medical University & Shandong Provincial Qianfoshan Hospital, Jinan, Shandong, China; c Department of Emergency Medicine, First Affiliated Hospital of Shandong First Medical University & Shandong Provincial Qianfoshan Hospital, Jinan, PR China; d Shandong Institute of Neuroimmunology, Jinan, Shandong, China; e Shandong Provincial Key Laboratory of Rheumatic Disease and Translational Medicine, Jinan, Shandong, China.

**Keywords:** bariatric surgery, nitrous oxide, subacute combined degeneration, vitamin B12 deficiency

## Abstract

Several studies have reported subacute combined degeneration (SCD) induced by nitrous oxide (N2O) abuse. However, few studies have reported that N2O-induced SCD recurred because of sleeve gastrectomy after neurological symptoms improved.

**Patient concerns:**

We report the case of an 18-year-old woman who developed paresthesia, weakness in 4 limbs, and an unstable gait after frequent, excessive N2O inhalation.

**Diagnosis:**

The patient was diagnosed as having SCD.

**Interventions and outcomes:**

Nineteen days after intravenous mecobalamin and supplementation with other kinds of vitamin B, her weakness and paresthesia resolved. However, 7 months after discharge, the patient experienced recurrence following sleeve gastrectomy. Blood biochemistry revealed low vitamin B12 levels. After a 22-day treatment, similar to the first hospitalization, her residual numbness and unsteady gait improved.

**Lessons:**

This case highlights that patients, especially those at high risk of vitamin B12 deficiency, undergoing sleeve gastrectomy require careful nutritional follow-up and routine monitoring of micronutrients such as vitamin B12 and homocysteine. Continuous vigilance is essential for patients with common and rare neurological complications.

## 1. Introduction

Subacute combined degeneration (SCD) is a potentially reversible disease that affects the posterior and lateral columns of the spinal cord and the peripheral nerves.^[1]^ It is mainly characterized by paresthesia, sensory ataxia, gait instability, and motor weakness.^[2]^ SCD is typically associated with vitamin B12 deficiency.^[3]^ Generally, the most common causes of vitamin B12 deficiency are inadequate intake, malabsorption, and compromised bioavailability.^[4]^

Nitrous oxide (N2O) is a widely used anesthetic agent.^[5]^ Nowadays, an increasing number of people use N2O as a recreational drug because of its effects on euphoria. Previous studies have shown that N2O abuse could cause neurotoxicity by interfering with vitamin B12 bioavailability and may cause neurological sequelae, such as axonal peripheral neuropathy and myelopathy.^[6]^ The clinical manifestations are similar to those of SCD with posterior and lateral column injuries.^[7]^

Over the past few decades, obesity has become a global public health issue. As traditional weight loss programs, such as diet and exercise, often fail to achieve long-lasting results, bariatric surgery is becoming an increasingly accepted and effective method for treating severe obesity.^[8]^ According to the International Federation for the Surgery of Obesity and Metabolic Disorders, 696,191 bariatric operations were performed worldwide in 2018.^[9]^ Bariatric surgery not only brings about favorable outcomes, but also resolves various metabolic diseases related to obesity, such as type 2 diabetes, obstructive sleep apnea syndrome, hypertension, and hyperlipidemia. However, despite satisfactory results, bariatric surgery still carries some risks, such as increasing the risk of vitamin B12 deficiency,^[10]^ leading to neurological complications.

Recently, sporadic cases of SCD in healthy young adults caused by N2O abuse have been described. However, few studies have reported a relapse of N2O-induced SCD due to bariatric surgery after the improvement of neurological symptoms. Herein, we present the case of a patient with recurrent SCD after bariatric surgery, drawing further attention to micronutrient deficiencies after bariatric surgery.

## 2. Consent

This study was approved by the ethics committee of the First Affiliated Hospital of Shandong First Medical University & Shandong Provincial Qianfoshan Hospital. Patient consent was obtained.

## 3. Case report

An 18-year-old woman presented to the neurology department with a 4-month history of paresthesia and weakness in four limbs.

She was a nonsmoker and rarely drank alcohol but described N2O abuse every week for 3 years. Four months ago, after taking ≈40 L of N2O, she developed numbness and weakness in her four limbs and difficulty in standing. Her gait was unstable and described as “walking on pillows.” Especially in a dark environment, her posture became more unstable. She had not inhaled N2O since she had difficulty in standing. Because of persistent symptoms, she was administered oral mecobalamin 500 µg three times a day. However, her residual symptoms prompted her to seek medical help. She was admitted to the general ward for further treatment.

Subsequent medical workup and auxiliary examination suggested abnormalities. On neurological examination, she had normal muscle strength in all 4 limbs, hyperreflexia in both lower limbs, and deep sensory loss in the right lower limb. In addition, Romberg sign and Babinski sign were positive, and the gait was unsteady.

Blood tests showed a normal red blood cell count, hemoglobin, mean corpuscular volume, and white cell count. The basic biochemistry, including that of urea and electrolytes, was unremarkable. Vitamin B12 level was higher than 2000 pg/mL (normal range: 200–900 pg/mL), with normal folate and homocysteine levels. Compound muscle action potential (CMAP) amplitudes decreased in both the common peroneal and bilateral tibial nerves. However, motor and sensory conduction velocities of the four limbs showed no abnormalities. The frequency of F-wave occurrence decreased. Somatosensory-evoked potentials were absent.

**Figure 1.**
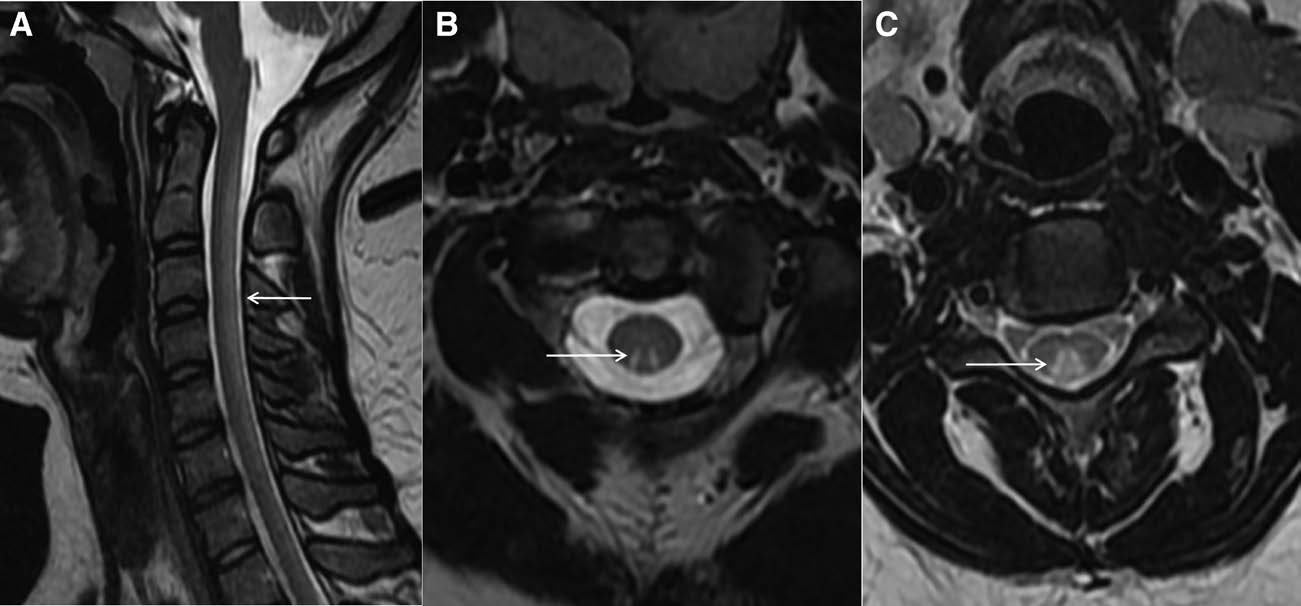
An 18-yr-old woman with subacute combined degeneration of the spinal cord due to nitrous oxide abuse in the first hospitalization. The sagittal T2-weighted images show a high signal along the posterior column of the cervical spinal cord (A, white arrows). Axial T2-weighted images demonstrate hyperintense signal in the dorsal columns of the cervical cord with the “inverted V” sign (B, white arrow) and the “triangle” sign (C, white arrow).

Magnetic resonance imaging (MRI) of the cervical spine demonstrated T2 hyperintensity involving the dorsal columns of the cervical spinal cord on the sagittal axis (Fig. 1A) and “inverted V” sign, “triangle” sign on the horizontal axis (Fig. 1B, C). These findings are consistent with SCD, which may have been caused by vitamin B12 inactivation due to N2O abuse. The patient was treated with intravenous mecobalamin (1 mg, once a day), oral vitamin B complex tablets (2 tablets, 3 times a day, containing vitamin B1 3 mg, vitamin B2 1.5 mg, vitamin B6 0.2 mg, nicotinamide 10 mg, calcium pantothenate 1 mg) and intramuscular vitamin B1 (1 mg, once a day) to prompt nerve repair.

After 19 days of therapy, the patient’s neurological function partially recovered, manifested as the disappearance of lower limb weakness and amelioration of numbness. The patient’s condition improved, and she was discharged successfully. The doctor ordered her to continue taking mecobalamin (500 µg) thrice a day. She stopped taking mecobalamin by herself when her symptoms further improved after discharge. She also discontinued N2O abuse. She was in a stable condition, with restored muscle strength and reduced numbness.

**Figure 2.**
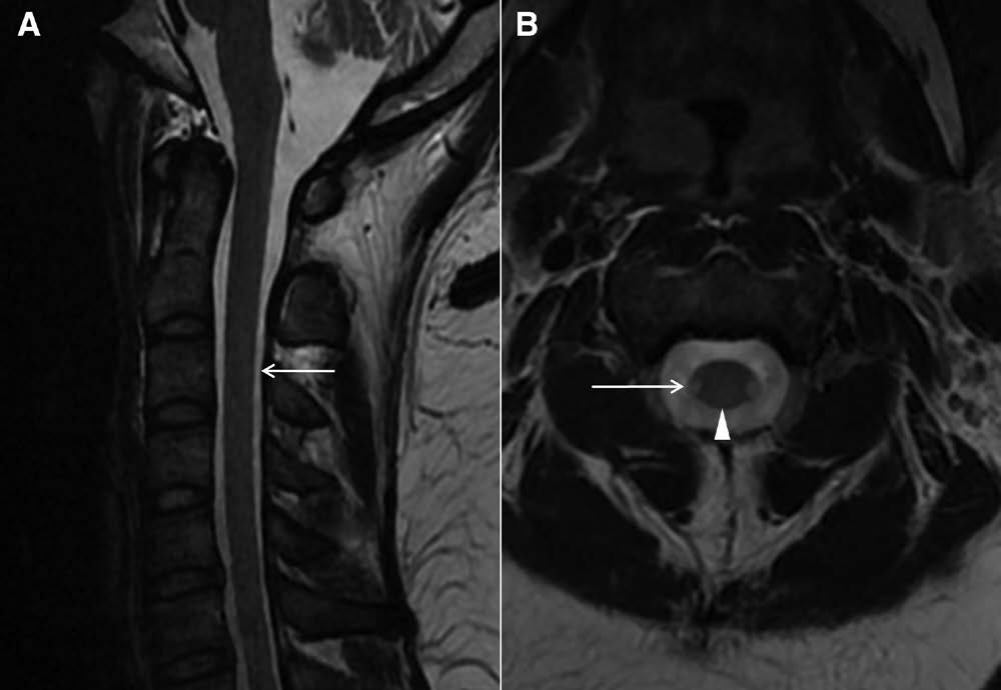
MRI 7 mo later. MRI of cervical cord shows disappeared hyperintense signal in posterior column (A, white arrow; B, triangular arrow). Axial T2 image indicates hyperintensity in the lateral column of the cervical cord (B, white arrow). MRI = magnetic resonance imaging.

Seven months after discharge, the patient presented to the neurology department again because of a 30-day history of worsening lower limb weakness, numbness in the distal extremities, and gait instability. Neurological examination revealed brisk reflexes in both lower limbs; Romberg sign, Babinski sign, and Chaddock sign were positive. Her vitamin B12 level was 215 pg/mL. MRI of the cervical spine showed that the posterior column lesions disappeared (Fig. 2A, B), but hyperintensity appeared in the lateral columns (Fig. 2B). All these findings are in line with SCD recurrence.

We further investigated the history to determine the cause of recurrence. At her first hospitalization, the patient’s weight was 123 kg. She tried to lose weight after discharge by dieting but failed. Her weight may decrease by 15 kg but rebound rapidly. She had undergone laparoscopic sleeve gastrectomy (SG) 40 days previously. Before the bariatric surgery, her weight was 125 kg (body mass index: 45.07). The gastric fundus and a large portion of the gastric body were resected along the greater curvature (15 cm from the pylorus and 2 cm from the cardia). She had 3-day fasting during the perioperative period, followed by a liquid diet before seeking medical help. Additional history taking revealed serum vitamin B12 was at 255.53 pg/mL and homocysteine raised at 138.4 µmol/L (normal range < 15 µmol/L) levels before surgery. When the patient was rehospitalized for limb weakness and gait instability, she received the same treatment as in the first hospital stay. She was discharged successfully after a 22-day treatment with alleviated numbness and weakness and improved unsteady gait. The patient was advised to continue taking methylcobalamin (500 µg) thrice a day.

Following discharge, the vitamin B12 level was requested but not performed because of the patient’s noncooperation. They were not repeated as they were unlikely to alter patient management. In the latest outpatient review, neurological examination was otherwise unremarkable. Clinical manifestation, relevant examination, and treatment at 2 hospitalizations are listed in Table 1.

**Table 1:** Comparison of clinical manifestation, relevant examination, and treatment at 2 hospitalizations.

	The first hospitalization	Rehospitalization for SCD
Possible trigger	Nitrous oxide abuse	Bariatric surgery
Clinical manifestation	Paresthesia and weakness in four limbs; unstable gait	Worsening lower limb weakness; numbness in the distal extremities; gait instability
Physical examination	Normal muscle strength; hyperreflexia in lower limbs; deep sensory loss in the right lower limb; Romberg sign and Babinski sign were positive	Brisk reflexes in both lower limbs; Romberg sign, Babinski sign, and Chaddock sign were positive
Vitamin B12 level (pg/mL)	>2000	215
Electrophysiological examination	Decreased compound muscle action potential amplitudes in the peroneal and bilateral tibial nerves; normal motor and sensory conduction velocities; reduced frequency of F-wave occurrence; absent somatosensory-evoked potentials	Not done
T2-weighted MRI of the cervical spine	T2 hyperintensity involving dorsal columns	Disappeared posterior column lesions; hyperintensity in lateral columns
Treatment	Intravenous mecobalamin; oral vitamin B complex tablets; intramuscular vitamin B1	Intravenous mecobalamin; oral vitamin B complex tablets; intramuscular vitamin B1
Treatment effect	Disappeared lower limb weakness and attenuated numbness	Alleviated numbness and weakness and improved unsteady gait
Treatment after hospital discharge	Took mecobalamin thrice a day, then discontinued when symptoms further improved	Took mecobalamin for long time

MRI = magnetic resonance imaging, SCD = subacute combined degeneration.

## 4. Discussion

Clinically, SCD begins with insidious subacute paresthesia in the hands and feet and gradually progresses to sensory loss, ataxia, and weakness.^[11]^ The clinical diagnosis is confirmed by low serum vitamin B12 and homocysteine levels (which should be measured in suspected cases with normal serum vitamin B12). This is supported by typical MRI findings, with or without abnormal nerve conduction studies.^[11,12]^

N2O abuse causes adverse neurological effects such as SCD, myelopathy, and generalized polyneuropathy.^[13]^ They may present with paresthesia, gait instability, and weakness.^[14,15]^ Heavy users are more prone to neurological sequelae than heavy users.^[14]^ Prolonged use of N2O leads to sustained interference with the vitamin B12 metabolic pathway, resulting in hematological and neurological dysfunction. N2O irreversibly oxidizes the cobalt ions of vitamin B12, rendering it inactive.^[16]^ It is not available as a coenzyme to convert homocysteine to methionine (methylcobalamin) or methyl-malonyl-coenzyme A to succinyl coenzyme A (adenosylcobalamin), leading to increased serum homocysteine levels.^[15,16]^ This sequence interrupts the methylation of myelin proteins, resulting in unstable myelin sheaths and demyelination, involving both the central and peripheral nervous systems.^[17]^

Here, we report the case of a patient who developed numbness, limb weakness, and gait instability after N2O abuse. The results of the physical examination, neuroimaging, and electromyography suggested lesions in peripheral neuropathy and spinal cord dorsal column. Because the patient took methylcobalamin by herself, the level of serum vitamin B12 was high and homocysteine was normal at the first hospitalization. Diagnosis of SCD was highly suggested combination of the history of N2O abuse before the onset of the disease. Intravenous mecobalamin and oral vitamin B complex tablets alleviated the symptoms, which was sufficient to establish the diagnosis of SCD. Although copper deficiency can manifest as myelopathy and peripheral neuropathy, mimicking SCD. The patient has a clear history of N2O abuse, and she recovered rapidly via supply of mecobalamin and other vitamin B instead of copper. The copper deficiency was excluded.

Currently, bariatric surgery is regarded as the most effective treatment option for severe obesity in adults. The most common bariatric surgeries performed were laparoscopic Roux-en-Y gastric bypass (LRYGB) and SG. Patients undergoing LRYGB or SG are at a high risk of vitamin B12 deficiency.^[18]^ Reports on the time at which vitamin B12 levels decrease after bariatric surgery are inconsistent. A previous study reported a decline in vitamin B12 within three months of weight loss surgery in some patients.^[19]^ Patients undergoing bariatric surgery have almost no gastric acid remaining in the gastric pouch, which greatly decreases the liberation of food-bound vitamin B12.^[20]^ Moreover, the production of intrinsic factor (IF) is reduced or absent in the bypassed stomach.^[21]^ Vitamin B12 malabsorption is potentiated by the delayed introduction of pancreatic enzymes into the distal jejunum. Thus, little or no IF is available to bind to the limited amount of vitamin B12 liberated from food or recycled with bile, thereby jeopardizing receptor-mediated absorption in the distal ileum. Previous studies have suggested that some patients with a normal range of serum vitamin B12 levels show symptoms of vitamin B12 deficiency. Elevated homocysteine levels can be evidence of vitamin B12 dysfunction.^[22,23]^ In the present case, the patient again developed limb weakness, numbness, and gait instability after the operation. Clinical and imaging outcomes suggested SCD recurrence. Further reviewing the literature, we did not find any reports of SCD recurrence within a short time (40 days) after SG. We consider the following reasons for this. First, the patient stopped taking mecobalamin when she felt better after the first discharge. We inferred that the spinal lesions did not recover fully and were sensitive to low-normal vitamin B12 levels. Second, dieting impaired vitamin B12 absorption, and the body had already consumed vitamin B12, which was reflected in the elevated homocysteine levels before SG. Third, a sudden reduction in food intake during the perioperative period and after surgery-induced SCD recurrence. Fourth, excision of gastric tissue further impaired the absorption of vitamin B12. However, the patient had a good recovery. She stated that eating a healthy balanced diet, giving up N2O, and supplying methylcobalamin played an important role.

Regrettably, the antiparietal antibody was not tested because the patient refused. Such as it is, this case underscores that doctors, especially surgeons, should pay attention to the monitoring and supplementation of vitamin B12 before and after bariatric surgery, especially for patients with underlying diseases caused by vitamin B12. At the same time, it reminds us that in addition to vitamin B12, timely detection and treatment of elevated homocysteine levels are equally crucial for SCD prevention.

## Author contributions

Data curation: Zhihua Si.

Formal analysis: Bing Yang.

Investigation: Wei Chen, Yanping Bi.

Project administration: Bing Yang.

Supervision: Zhihua Si, Bing Yang.

Writing—original draft: Wei Chen.

Writing—review & editing: Bing Yang.
